# Terminal Complement Pathway Deficiency in an Adult Patient with Meningococcal Sepsis

**DOI:** 10.1155/2022/9057000

**Published:** 2022-05-23

**Authors:** F. Staels, W. Meersseman, P. Stordeur, K. Willekens, S. Van Loo, A. Corveleyn, I. Meyts, G. Meyfroidt, R. Schrijvers

**Affiliations:** ^1^Department of Microbiology, Immunology and Transplantation, Laboratory of Adaptive Immunology, KU Leuven, Leuven, Belgium; ^2^Department of Microbiology, Immunology and Transplantation, Allergy and Clinical Immunology Research Group, KU Leuven, Leuven, Belgium; ^3^Department of General Internal Medicine, University Hospitals Leuven, Leuven, Belgium; ^4^Department of Microbiology, Immunology and Transplantation, Laboratory of Clinical Infectious and Inflammatory Disease, KU Leuven, Leuven, Belgium; ^5^Belgian National Reference Center for the Complement System, Laboratory of Immunology, LHUB-ULB, Université Libre de Bruxelles, Brussels, Belgium; ^6^Center for Human Genetics, University Hospitals Leuven, Leuven, Belgium; ^7^Department of Microbiology, Immunology and Transplantation, Laboratory for Inborn Errors of Immunity, KU Leuven, Leuven, Belgium; ^8^Department of Pediatrics, University Hospitals Leuven, Leuven, Belgium; ^9^Department of Cellular and Molecular Medicine, Laboratory of Intensive Care, KU Leuven, Leuven, Belgium; ^10^Department of Intensive Care, University Hospitals Leuven, Leuven, Belgium

## Abstract

The complement system is an essential part of our innate immune system. Three enzymatic activation pathways are described, all converging into a common terminal pathway which causes lysis of the target cell. Late complement deficiencies (LCDs) are typically diagnosed in children or adolescents with invasive meningococcal disease (IMD). However, IMD can also be a first manifestation in adulthood and should prompt for the evaluation of the LCD. We report the case of a young adult with IMD who was found to have a LCD, caused by a compound heterozygous mutation in *C6*. His vaccination status was optimized and prophylactic antibiotic treatment was initiated. By means of this case, we would like to raise awareness of underlying LCD in (young) adults presenting with IMD by *N*. *meningitidis*. Screening for complement deficiencies after IMD, followed by genetic testing, can be lifesaving and allows for genetic counselling. In addition, we discuss the diagnosis and treatment of LCD.

## 1. Introduction

**Figure 1:**
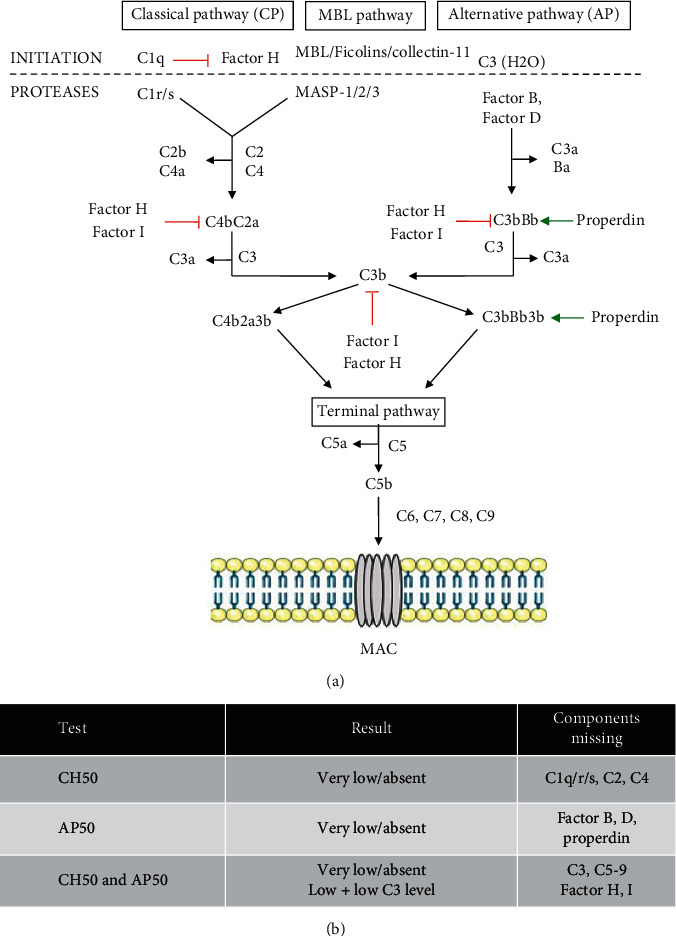
(a) Activation of the CP, AP, and LP. In the CP, C1q binds antigen-antibody complexes. After binding, C1r and C1s proteases cleave C4 and C2 resulting in the formation of a C4b2a (CP C3 convertase). This C3 convertase cleaves C3 into C3a and C3b forming the C4b2a3b (CP C5 convertase) which initiates the common terminal pathway and the formation of a MAC complex. In the AP, spontaneous hydrolysis of C3 forms C3(H2O) which associates with factor B and D to form the C3bBb (AP C3 convertase). Similar to CP, C3 is cleaved leading to the formation of a C3bBb3b complex (AP C5 convertase) and subsequent activation of the terminal pathway. The LP occurs through different initiators that bind high-density sugars of pathogens. After this binding, LP-associated serine proteases (MASP) form a complex with the recognition molecules to allow the cleavage of C4 and C2, similar to the CP. Properdin functions as a stabilizer of the membrane bound C3bBb and C3bBb3b. Factor H functions as a negative regulator of CP and AP by blocking formation of the AP C3 convertase, cleavage, and inactivation of C4b and preventing C1q binding to its ligands. Factor I mediates the cleavage and inactivation of C3b and C4b, blocking the formation of the C3 convertase. Red arrows indicate inhibitory routes, and green arrows indicate stabilizing routes. (b) Interpretation of CH50 and AP50 values in the diagnosis of complement deficiencies. Further testing for individual components in the complement pathway should be performed depending on this result.

The complement system is part of our innate immunity, functioning as a first line of defense against bacterial infections. It is organized in three pathways: the classical pathway (CP), alternative pathway (AP), and lectin pathway (LP) [1, 2]. These pathways, initiated through different mechanisms, converge to a common terminal pathway (Figure 1(a)). In the terminal pathway, C5 convertase cleaves C5 to C5b which recruits C6, C7, C8, and C9 to form the membrane-attack-complex (MAC) [1, 2]. This complex forms a pore in the membrane, leading to lysis of the pathogen. Complement deficiencies in the early pathway are associated with diverse autoimmune diseases such as systemic lupus erythematosus (SLE) [3] with or without infectious susceptibility (mostly by encapsulated bacteria). In contrast, deficiencies in the terminal pathway (LCD) are typically seen in a pediatric population presenting with invasive meningococcal disease caused by *Neisseria meningitidis* (IMD) [4, 5]. However, IMD can also manifest in adulthood unmasking an underlying LCD [6]. We report the case of an 18-year-old man who presented with IMD and was confirmed to have a LCD due to a compound heterozygous mutation in *C6* resulting in absent C6 levels.

## 2. Case Presentation

**Figure 2:**
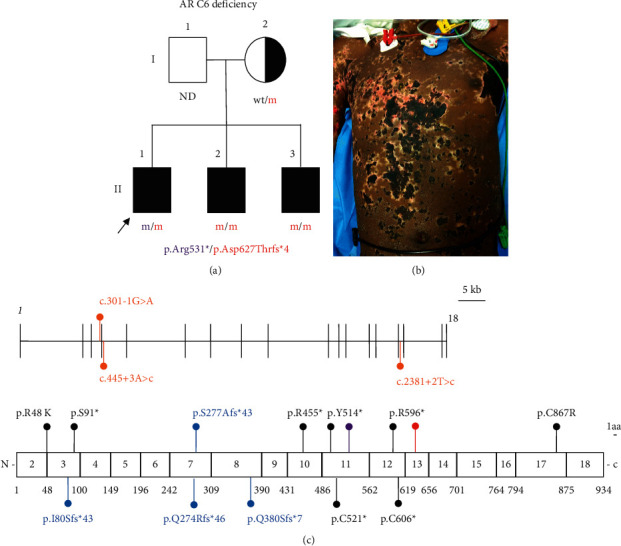
(a) Family pedigree and genotype. Compound heterozygosity was found in the index patient for c.1879delG, p.Asp627Thrfs^*∗*^4 (classified as known pathogenic) and c.1591C > T, p.Arg531^*∗*^ (predicted as a complete loss of function, unreported) and homozygosity was found in the siblings for c.1879delG. The mother was carrier for c.1879delG. Genetic material of the father was not accessible (familial reasons) and no further exploration on the inheritance mechanism was performed. (b) Clinical presentation of purpura fulminans at second admission. (c) Top: *C6* gene structure, containing 18 exons, and pathogenic intronic mutations (*n* = 3) affecting the splicing of *C6* are shown (orange); bottom: C6 protein structure encoded by the corresponding exons (italic) with known pathogenic missense/nonsense mutations in black (*n* = 8) and frameshift mutations in blue (*n* = 4), source: Human Gene Mutation Database. The position of the pathogenic mutations in the index patient is depicted (red, purple).

An 18-year-old Surinam patient (Figure 2(a)), born to non-consanguineous parents, presented with high grade fever (39.9°C), vomiting, diffuse myalgia, headache, and petechial bleeding on the arms and trunk. Clinical evaluation showed signs of meningeal irritation and skin petechiae. Laboratory analysis showed elevated inflammatory parameters (CRP 360 mg/L, reference <5 mg/L; neutrophils 11.3 × 10^3^/*μ*L, reference 1.5–5.7 × 10^3^/*μ*L). Blood cultures were negative, and the patient received empirical treatment with ceftriaxone (2 g BID). Lumbar puncture was not performed due to anticoagulation for an unprovoked pulmonary embolism 5 months earlier. The patient rapidly improved under antibiotic treatment, suggesting an infectious cause of meningitis.

Nine months later, he was readmitted to the intensive care unit with diffuse myalgia, fever (39.6°C), abdominal pain, headache, and somnolence. Blood analysis showed elevated inflammatory parameters (CRP 164 mg/L, neutrophils 6.2 × 10^3^/*μ*L), thrombopenia (90 × 10^3^/*μ*L, reference platelets 150–400 × 10^3^/*μ*L), impaired renal function (creatinine 1.81, reference 0.72–1.21 mg/dL), and a metabolic acidosis (arterial pH 7.31; lactate 99.1 mg/dL, reference 5.5–14.4 mg/dL; bicarbonate 13.4 mmol/L, reference 21–28 mmol/L; pCO2 27.4 mmHg, reference 35–48 mmHg). Blood cultures were positive for *N*. *meningitidis*. Transthoracic echocardiography showed a decreased left ventricular ejection fraction (25%, reference 55–70%). Fluid resuscitation and vaso- and inopressors were initiated for a combined septic and cardiogenic shock. Because of progressive hypoxic respiratory failure, mechanical ventilation was initiated. Antibiotics (ceftriaxone 2 g BID and amikacin 1 g once) were initiated. However, despite early treatment initiation, he progressively developed purpura fulminans (Figure 2(b)) with diffuse intravascular coagulation and rhabdomyolysis of his limbs complicated by a compartment syndrome necessitating amputation of his left upper arm and multiple fasciotomies of other limbs. After five months on the intensive care unit, the patient was discharged for intensive rehabilitation. The occurrence of recurrent meningitis, once confirmed due to *N*. *meningitidis* serogroup Y, raised suspicion of a complement deficiency. Functional testing revealed a decreased classical and alternative pathway activity, with normal C3 and C4 suggesting a LCD (Table 1). Genetic testing revealed the presence of compound heterozygous mutations in *C6* (Figure 2(a)). The C6 level was proved to be deficient (Table 1), confirming the pathogenicity of the mutations. The screening of siblings demonstrated that both were affected. The patient and siblings received conjugated meningococcal vaccines for serotypes A/C/W/Y and B, pneumococci, and *H*. *influenzae* type B. Prophylactic antibiotics (azithromycin 3x/week) were started.

## 3. Discussion

Establishing a diagnosis of an inherited complement deficiency is crucial in patients with IMD. Up to 20% of the patients suffering from IMD lack any of the terminal complement components, or properdin, the latter leading to an X-linked deficiency of the alternative pathway [7]. Meningococcal isolates causing IMD in LCD patients commonly belong to minor or uncommon serogroups, especially serogroup Y, although no specific correlation between LCD and a certain capsular group was found [5, 8]. In this case, a complement deficiency was suspected after the second presentation with IMD with isolation of *Neisseria meningitidis* serogroup Y. Screening for complement deficiencies can be performed by determination of the functional activity of the CP (CH50), the AP (AH50), and the MBL pathway [9]. The gold standard for measuring the CH50 is based on the minimal dilution of serum that causes lysis of 50% of sheep erythrocytes. Nowadays, in most laboratories, CH50 is measured using an automated liposome-based immunoassay [10]. In this assay, sensitized liposomes containing glucose-6-phosphate dehydrogenase (G6PD) are mixed with serum together with a substrate containing G6P, nicotinamide adenine dinucleotide, and anti-DNP antibody. Upon liposome lysis, an enzymatic colorimetric reaction occurs which is proportional to the classical complement activity. For measuring AH50, the gold standard assay is similar to the CH50 gold standard approach, but with the use of rabbit erythrocytes (in contrast to sheep erythrocytes, their surface promotes more AP activation) and dilution of serum in a diluent containing blockers of the CP and MBL pathway. Nowadays, this is replaced by an enzyme-linked immunosorbent assay (ELISA) where a plate coated with zymosan (activator of the AP pathway) is incubated with a serum sample and the terminal complement complex (TCC, soluble C5b-9) is measured using an anti-TCC conjugated antibody [10]. LP is not routinely incorporated in the first screening, but can be tested by incubating serum of the patient on a mannan coated plate. Similar to the AP assay, TCC is measured using ELISA [10]. The interpretation of CH50 and AP50 results is shown in Figure 1(b). If CP components are missing (C1q/r/s, C2 or C4), CH50 will be very low or absent with a normal AP50. If AP components are missing (factor B, D or properdin), AP50 will be very low or absent with a normal CH50. In case both AH50 and CH50 are very low or absent, a C3 defect or LCD (C5–9) should be suspected or in rare cases, factor H or I deficiency. After determination of CH50 and AP50, further quantification of individual complement levels can be performed depending on the results. In our patient, both CH50 and AP50 were very low (CH50) or absent (AH50) with normal C3 levels (Table 1). Further quantitative determination of the late components (C5–9) revealed that C6 levels were undetectable (Table 1). Both whole exome and targeted sequencing of the *C6* gene in the index patient revealed a compound heterozygous mutation c.1879delG, p.Asp627Thrfs^*∗*^4 (classified as known pathogenic) and c.1591C > T, p.Arg531^*∗*^ (predicted as a complete loss of function, unreported). Both his siblings were screened for CH50 and AP50 activity and also found to be defective caused by absent C6 levels, while his mother had normal CH50 and AP50 activity. Surprisingly, his siblings had the c.1879delG, p.Asp627Thrfs^*∗*^4 in homozygosity. Genetic material of the father was not accessible (for familial reasons) and therefore no further exploration on the inheritance mechanism was performed. Before genetic confirmation, an autosomal dominant inheritance pattern could have been conceived. However, dominant complement disorders have hitherto only been associated with atypical hemolytic uremic syndrome in which the risk for meningococcal infections is deemed low, unless after eculizumab treatment [11]. The abnormal CH50 ruled out the only X-linked recessive complement deficiency (properdin deficiency) in the 2 brothers. Furthermore, no defects in *CFP* (encoding properdin) were found in whole exome sequencing data from the index patient. C6 deficiency is caused by autosomal recessive loss-of-function mutations in *C6*, with 16 mutations in 65 cases reported (Figure 2(c)). The treatment of LCD includes optimization of vaccination status (tetravalent meningococcal vaccine A/C/W/Y and the recently available vaccine against serogroup B, *H*. *influenzae* type B, and pneumococcal vaccines), even in asymptomatic patients. Tetravalent meningococcal vaccine and vaccine against serogroup B are available in Belgium but are not included in the routine vaccination scheme and not reimbursed. Efficacy of meningococcal vaccination was assessed in a cohort of 45 Russian patients with LCD, of whom 31 were vaccinated, with a follow up of 3–8 years [12]. Meningitis episodes decreased significantly in the vaccinated versus non-vaccinated group, but still occurred (6 episodes in 4 patients throughout follow-up). Importantly, half of these new meningitis episodes were caused by serogroup B or non-serogroupable meningococci, suggesting that additional vaccination to serogroup B might improve protection against meningococcal infection. Since vaccination is not providing complete protection, prophylactic antibiotics are routinely given to patients with LCD [13]. Randomized controlled trials studying the effectiveness of prophylactic antibiotics are lacking because of the rarity of LCD. A prospective study in South Africa with 40 C6 deficient patients reported beneficial effects of monthly benzathine penicillin G in high-risk patients (≥2 episodes of meningococcal infection) on the occurrence of new meningococcal infections [14]. However, the patients in this study were not vaccinated and the control group was not given a placebo. The role of prophylactic antibiotics in this population, in the context of changing epidemiology and increasing vaccination coverage, has not been properly studied. At this moment, the latest recommendation from the European Society of Immunodeficiency Diseases (ESID) is to offer antibiotic prophylaxis based on risk stratification (e.g., living in endemic areas or having a high-risk profession such as nursery care) [15]. In our case, taking into account that these recommendations are not based on RCTs or recent prospective studies, we discussed the options together with the patient and his affected relatives and decided to give prophylactic antibiotics (azithromycin 3 × 250 mg/week, based on previous studies showing efficacy of azithromycin against *Neisseria meningitidis* isolates [16], stable serum concentrations, and convenience of the regimen) because of the severity of the infection and the psychological impact.

## Figures and Tables

**Table 1:** Summary of demographic, clinical, and complement levels in the index patient and relatives (ND: not determined).

	II.1 (index)	I.2	II.2	II.3
Age	19 years	38 years	18 years	11 years
Infections	Meningococcal sepsis	—	—	—
Classical complement pathway activity (%, 66–113)	<12.5	98.1	<12.5	<12.5
Alternative complement pathway activity (%, 20–60)	0	24	0	0
C3 (7.9–15.2 mg/dl)	18.1	ND	10.7	14.8
C4 (1.6–3.8 mg/dl)	3.8	ND	1.3	2.2
C5 (1.1–25 mg/dl)	34.5	ND	ND	ND
C6 (>4.5 mg/dl)	<0.5	ND	<0.5	<0.5
C7 (>5.5 mg/dl)	8.9	ND	ND	ND
C8 (>11.2 mg/dl)	19.4	ND	ND	ND
C9 (>12.5 mg/dl)	17.5	ND	ND	ND
